# Deep Brain Stimulation of the Forel’s Field for Dystonia: Preliminary Results

**DOI:** 10.3389/fnhum.2021.768057

**Published:** 2021-11-29

**Authors:** Shiro Horisawa, Kotaro Kohara, Masato Murakami, Atsushi Fukui, Takakazu Kawamata, Takaomi Taira

**Affiliations:** Department of Neurosurgery, Neurological Institute, Tokyo Women’s Medical University, Tokyo, Japan

**Keywords:** Forel’s field, pallidothalamic tract, deep brain stimulation, dystonia, globus pallidus internus

## Abstract

The field of Forel (FF) is a subthalamic area through which the pallidothalamic tracts originating from the globus pallidus internus (GPi) traverse. The FF was used as a stereotactic surgical target (ablation and stimulation) to treat cervical dystonia in the 1960s and 1970s. Although recent studies have reappraised the ablation and stimulation of the pallidothalamic tract at FF for Parkinson’s disease, the efficacy of deep brain stimulation of FF (FF-DBS) for dystonia has not been well investigated. To confirm the efficacy and stimulation-induced adverse effects of FF-DBS, three consecutive patients with medically refractory dystonia who underwent FF-DBS were analyzed (tongue protrusion dystonia, cranio-cervico-axial dystonia, and hemidystonia). Compared to the Burke-Fahn-Marsden Dystonia Rating Scale-Movement Scale scores before surgery (23.3 ± 12.7), improvements were observed at 1 week (8.3 ± 5.9), 3 months (5.3 ± 5.9), and 6 months (4.7 ± 4.7, *p* = 0.0282) after surgery. Two patients had stimulation-induced complications, including bradykinesia and postural instability, all well controlled by stimulation adjustments.

## Introduction

Deep brain stimulation (DBS) of the globus pallidus internus (GPi) is the most widely used surgical treatment for medically refractory dystonia, and its effects have been validated (Kupsch et al., 2006; Volkmann et al., 2014). However, stimulation that spreads to surrounding structures or of the GPi itself can cause not only visual and motor complications but also Parkinsonism, including bradykinesia, postural instability, and gait disturbance (Berman et al., 2009; Zauber et al., 2009; Schrader et al., 2011; Huebl et al., 2015; Rusz et al., 2018; Koeglsperger et al., 2019). These stimulation-induced adverse effects have remained unresolved due to the anatomical location of the GPi and may suppress the optimal clinical effects of GPi-DBS (Schrader et al., 2011).

The field of Forel (FF) is a subthalamic area traversed by various types of white matter (Neudorfer and Maarouf, 2018). The pallidothalamic tract, including the ansa lenticularis and lenticular fasciculus, both originating from the globus pallidus internus (GPi), merge and become thalamic fasciculus at the FF and reach thalamic subnuclei (Neudorfer and Maarouf, 2018). The FF was used as a stereotactic surgical target (ablation and stimulation) to treat cervical dystonia in the 1960s and 1970s (Loher et al., 2004). We recently reported a significant effect of radiofrequency ablation on dystonia, whereby intraoperative stimulation of the FF did not evoke any sensory or motor response (Horisawa et al., 2019a). Based on our experience and previous studies, we expected deep brain stimulation (DBS) of the FF (FF-DBS) to significantly improve dystonia without intractable stimulation-induced adverse effects. We report preliminary results of three patients with dystonia who underwent FF-DBS.

## Patients and Methods

The ethics committee of the Tokyo Women’s Medical University approved the study. Written informed consent was obtained from all patients.

### Patients

Three patients with dystonia underwent FF-DBS after failed thalamotomy or pallidotomy. The definition of failed surgery was defined as < 50% improvement of Burke-Fahn-Marsden Dystonia Rating Scale (BFMDRS) after surgery. One patient had tongue protrusion dystonia, another had left hemidystonia with right-hand tremor, and the last had tardive cranio-cervico-truncal dystonia. Their detailed clinical characteristics are shown in Table 1. The mean age at onset was 31.3 ± 3.3 years, and the mean disease duration was 5.8 ± 5.7 years. Case 1 patient with tongue protrusion dystonia and Case 2 patient with tardive cranio-cervico-truncal dystonia received bilateral pallidotomy resulting in temporary improvements. Case 3 patient initially presented left-hand tremor and received right ventral-intermediate (vim) thalamotomy, which resulted in complete resolution of tremor. Two years after the right thalamotomy, left hand and foot tremor with hand stiffness developed. Right ventro-oral (vo) and vim thalamotomy was performed. Despite complete symptom improvement, symptoms recurred 3 months after the right vo-vim thalamotomy. As additional ablative surgeries may have induced severe irreversible complications in those three patients, we selected DBS treatment. Additionally, we were concerned about the possibility that the treatment targets already ablated (GPi, Vo, and Vim) were not effective for dystonia improvement. Based on our experience showing that patients with dystonia receiving ablation of pallidothalamic tracts at FF showed significant improvement of dystonia, we selected the FF as an alternative surgical target of DBS. The absence of bradykinesia, gait disorder, and postural instability was confirmed in all patients.

**TABLE 1:** Patient characteristics.

		Dystonia					
Patient	Sex	Etiology	Distribution	Symptoms	Age at onset (year)	Disease duration (year)	Previous failed treatment
1	Male	Primary	Tongue	Tongue protrusion	29	5	Bilateral pallidotomy
2	Male	Tardive	Craniocervical-trunk	Blepharospasm, retrocollis	35	14	Bilateral pallidotomy, botulinum toxin injection
3	Male	Primary	Left hand, Left foot	Left hand clenched fist, left foot inversion	33	2	Botulinum toxin injection, thalamotomy

### Surgical Procedures

Cases 1 and 2 underwent simultaneous bilateral FF-DBS, and one patient (Case 3) underwent simultaneous right FF-DBS with left vim-DBS for tremor. All patients have previous failed pallidotomy or thalamotomy.

The implantation of directional DBS electrodes (Boston Scientific, Marlborough, MA, United States) was conducted under local anesthesia. T1-weighted axial and T2-weighted axial/coronal magnetic resonance imaging (MRI) were used to determine the stereotactic target. Tractography was not available due to the difficulty visualizing pallidothalamic fibers passing through FF. The FF target was set at 8–10 mm lateral, and 1.0–2.5 mm inferior and 0.5 mm posterior to the mid-commissure point, adjusted according to the location of the subthalamic nucleus and mammillothalamic tract. The mammillothalamic tract (MTT) as the medial boundary and STN as the inferior boundary are key structures to confirm the FF location. Both structures were clearly visualized as low-intensity areas on T2-weighted MRI. Macrostimulation (130 Hz, 100 μs, 2–3 V) with an external neurostimulator (Medtronic model 3625) did not elicit any sensorimotor response. We did not use microelectrode recording. An implantable pulse generator (Boston Scientific, Vercise Gevia) was placed under general anesthesia after electrode placement on the same day. A postoperative head CT scan was performed immediately after surgery to confirm the absence of hemorrhagic complications, and electrode location was confirmed using Brainlab Elements software (BrainLab, Munich, Germany).

### Measures

To evaluate dystonia, the Burke-Fahn-Marsden Dystonia Rating Scale-Movement Scale (BFM-MS) was completed before surgery, and 1 week, 3 months, and 6 months postoperatively. Stimulation-induced complications were also evaluated.

### Statistical Analysis

The data were considered non-parametric, and Wilcoxon’s signed-rank test was used to compare the BFM-MS score between before surgery and 6 months postoperatively. Differences with *p*-value < 0.05 were considered statistically significant. All statistical analyses were performed using JMP (version 13.0.0., SAS Institute). Data are represented as mean ± standard deviation.

## Results

**FIGURE 1:**
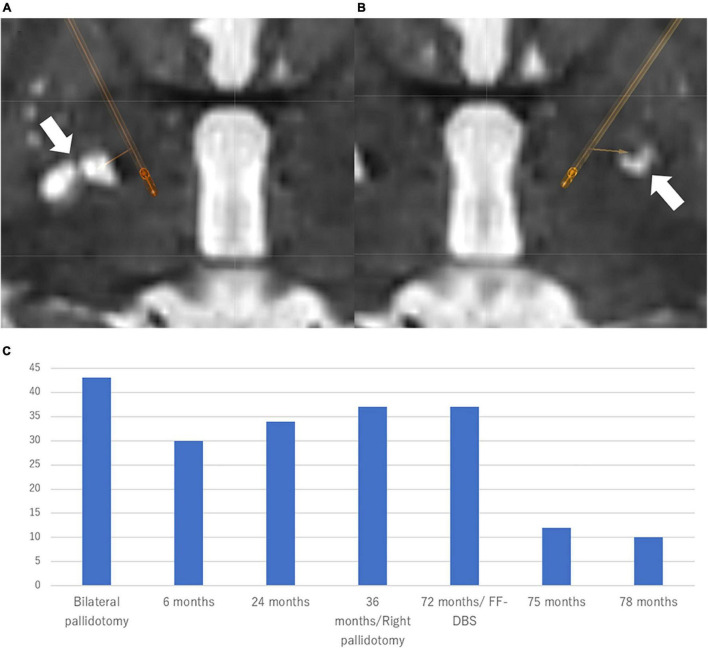
Location of the electrodes and clinical course in Case 1 Patient. The electrodes were simulated by Brainlab elements using postoperative CT scan fused with preoperative T2-weighted MRI. **(A)** Right-side stimulation: 10-(8%)/11-(6%)/12-(6%)/13-(28%)/14-(26%)/15-(26%)/C +, 80 μs, 159 Hz, 1.5 mA. **(B)** Left-side stimulation: 5-(34%)/6-(33%)/7-(33%)/C +, 80 μs, 159 Hz, 2.5 mA directional DBS electrodes (Boston Scientific, Marlborough, MA, United States) were used. **(C)** The patient experienced a 91.7% improvement of BFMDRS at 6 months after the bilateral Forel’s field DBS. BFMDRS: Burke-Fahn-Marsden dystonia rating scale (range: 0–120) DBS, deep brain stimulation.

**FIGURE 2:**
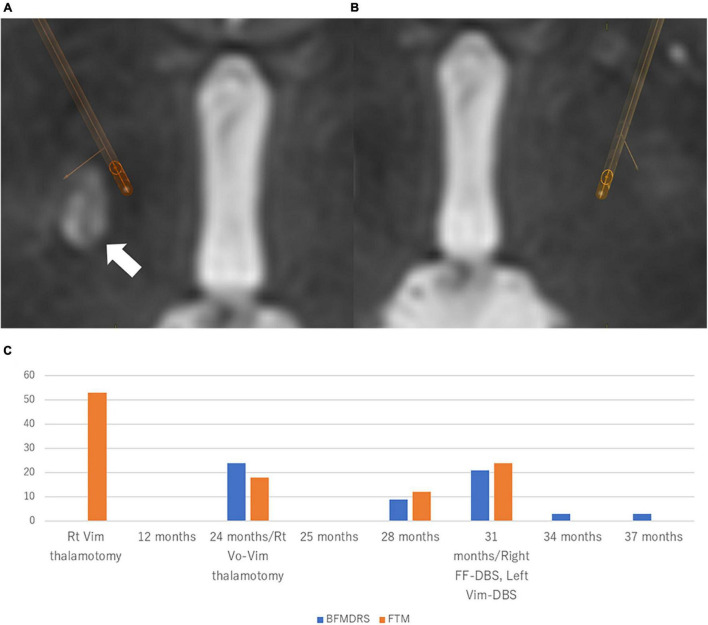
Location of the electrodes and clinical course in Case 2 Patient. The electrodes were simulated by Brainlab elements using postoperative CT scan fused with preoperative T2-weighted MRI. **(A)** Right-side stimulation: 13-(24%)/14-(23%)/15-(23%)/16-(30%)/C +, 130 μs, 179 Hz, 2.0 mA. **(B)** Left-side stimulation: 2-(20%)/3-(20%)/4-(20%)/5-(14%)/6-(13%)/7-(13%)/C +, 130 μs, 179 Hz, 2.0 mA. White arrow indicates the lesions of pallidotomy. Directional DBS electrodes (Boston Scientific, Marlborough, MA, United States) were used. **(C)** The patient experienced a 73.0% improvement of BFMDRS at 6 months after the bilateral Forel’s field DBS. BFMDRS, Burke-Fahn-Marsden dystonia rating scale (range: 0–120). DBS, deep brain stimulation.

**FIGURE 3:**
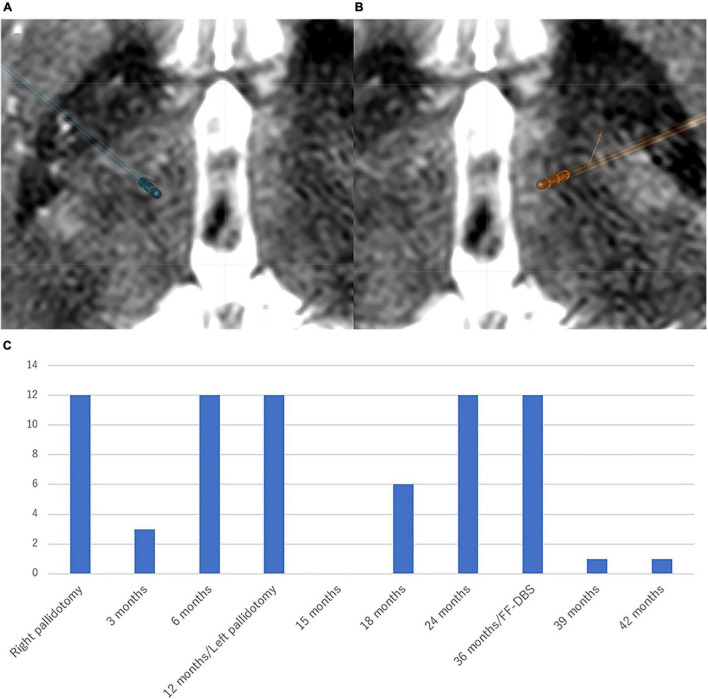
Location of the electrodes and clinical course in Case 3 Patient. The electrodes were simulated by Brainlab elements using postoperative CT scan fused with preoperative T2-weighted MRI. **(A)** Right-side stimulation (FF): 13-(24%)/14-(23%)/15-(23%)/16-(30%)/C +, 100 μs, 100 Hz, 2.5 mA. White arrow indicates the lesion of thalamotomy. **(B)** Left-side stimulation (vim): 2-(34%)/3-(33%)/4-(33%)/C +, 100 μs, 100 Hz, 2.5 mA. Directional DBS electrodes (Boston Scientific, Marlborough, MA, United States) were used. **(C)** The patient first received right vim thalamotomy for left-hand tremor. After 24 months post-surgery, tremor and dystonia developed in the left side of the body, which were well improved by right vim-vo thalamotomy. However, left-sided dystonia and right-sided tremor were newly developed 4 months after the second thalamotomy. Right Forel’s field DBS for left-sided dystonia and left vim-DBS for right-sided tremor were performed, which led to complete resolution of tremor and 85.7% improvement of BFMDRS at 6 months after the DBS surgery. BFMDRS, Burke-Fahn-Marsden dystonia rating scale (range: 0–120). FTM, Fahn-Tolosa-Marin tremor rating scale (range: 0–144). DBS, deep brain stimulation. Vim, ventral intermediate nucleus. Vo, ventro-oral nucleus.

The clinical outcomes are shown in Table 2. Stimulation began the day after surgery in all patients. Compared to the BFM-MS scores before surgery (23.3 ± 12.7), improvements were observed at 1 week (8.3 ± 5.9), 3 months (5.3 ± 5.9), and 6 months (4.7 ± 4.7, *p* = 0.0282) after surgery. Two patients showed significant stimulation-induced complications. Case 1 Patient had significant right-side bradykinesia and an unsteady gait, which were resolved by stimulation adjustments without dystonia deterioration. Case 3 Patient had an unsteady gait and postural instability, which were also resolved by stimulation adjustments without dystonia deterioration. No other stimulation-induced adverse events were found. The electrode location at active contact level in all three patients was confirmed by Brainlab Elements software (Figures 1A,B, 2A,B, 3A,B). The time course of BFM-MS with surgical interventions in all patients is shown in Figures 1C, 2C, 3C.

**TABLE 2:** Clinical outcomes and stimulation details.

	BFMDRS-MS					Stimulation settings		Location of active contacts	
Patient	Pre	1 week	3 months	6 months	% improvement	Rt	Lt	Rt (X, Y, Z)	Lt (X, Y, Z)
1	12	1	1	1	91.70%	80 μs, 160 Hz, 1.5 mA	80 μs, 160 Hz, 2.5 mA	(10.6, −1, 1)	(10, 0.9, 0)
2	37	15	12	10	73.00%	130 μs, 180 Hz, 2.0 mA	130 μs, 180 Hz, 2.0 mA	(12.6, 0, 0)	(11, −1, 1)
3	21	3	3	3	85.70%	100 μs, 100 Hz, 2.5 mA		(12.0, 0, 1.3)	

*BFMDRS-MS, Burke-Fahn-Marsden Dystonia Rating Scale-Movement Scale.*

## Discussion

This preliminary report showed a 79.8% improvement of dystonia measured by the BFM-MS 6 months after FF-DBS. Two patients developed stimulation-induced complications, which were well controlled by adjusting stimulation settings without any deterioration of dystonia. The locations of active contacts were below the thalamus and above the subthalamic nucleus.

One of the pathophysiological bases for dystonia is abnormality of the cortico-basal ganglia-thalamo-cortical circuit (CBTC) (Vitek, 2002b). Symptomatic improvements induced by pallidal DBS were related to an enhancement of the activity of prefrontal or frontal cortico-basal ganglia-thalamo-cortical circuit in PET study. GPi-DBS is considered to redress the associated circuit abnormality, which results in symptomatic improvement (Fukuda et al., 2002; Mure et al., 2012). The pallidothalamic tract, which originates from the GPi, is a constituent pathway of the CBTC and reaches the thalamic nuclei through the FF (Gallay et al., 2008). Our recent study revealed that radiofrequency ablation of pallidothalamic tract on the FF significantly improved dystonia (Horisawa et al., 2019a,b). Recent studies suggested that the mechanism of action of DBS is functional blockade of information transmission including pathological information, which is called informational lesion (Grill et al., 2004; Chiken and Nambu, 2013). The informational lesion induced by DBS overrides the impact of pathological basal ganglia activity on downstream targets (Wichmann and DeLong, 2016). Both pallidal and thalamic DBS (ventro-oral nucleus, downstream of GPi) are effective for dystonia (Kupsch et al., 2006; Fukaya et al., 2007; Cho et al., 2009; Volkmann et al., 2014). Pallidothalamic tracts including ansa lenticularis (AL) and lenticular fasciculus (LF) connect GPi with ventro-anterior and ventro-lateral nucleus including ventro-oral nucleus (Gallay et al., 2008). AL and LF merge at FF H and ascend to thalamic nuclei through FF H1 (Gallay et al., 2008). Electrical stimulation on pallidothalamic tract at FF H1 may block the pathological information from basal ganglia output, thus leading to symptomatic improvements.

Subthalamic area was used as a stereotactic surgical target to treat cervical dystonia in the 1960s and 1970s (Loher et al., 2004). Hassler and Dieckmann reported efficacy of combined ablation of FF and ventro-oral internus nucleus for cervical dystonia (Hassler and Dieckmann, 1970). Zona incerta (ZI) is a subthalamic gray matter structure whose functional role is not well established. Mundinger et al. (1972) reported a better effect of combined ZI ablation including the FF than the GPi and ventro-oral internus nucleus in the treatment of cervical dystonia. Subsequently, Mundinger (1977) confirmed that electrical stimulation of ZI including FF improved cervical dystonia. The anatomical location of ZI is superior to the subthalamic nucleus and is situated between thalamic fasciculus and lenticular fasciculus. Due to the extreme close spatial location between the ZI and the FF (pallidothalamic fibers), it is difficult to distinguish which target contributed more to the symptomatic improvement. DBS of the dorsal border of the STN and ZI close to the Forel’s field H1 has been reported to improve cardinal motor symptoms, including drug-induced dyskinesia in PD (Voges et al., 2002; Alterman et al., 2004; Li et al., 2021). It has been established that the GPi-DBS is the effective procedure for dystonia. Compared to FF, GPi is the largest structure, including motor, limbic and associative territories. The optimal GPi location for the treatment of dystonia is the posteroventrolateral part, that is the motor portion, as well as the main output region of pallidal efferents to the thalamus (pallidothalamic tracts), as pallidothalamic tracts course through the FF finally entering the nucleus of the thalamus. Therefore, both electrical stimulation of the posteroventrolateral part of GPi and pallidothalamic tracts at FF share common treatment effects on dystonia by modifying cortico-basal ganglia-thalamo-cortical circuits through neuromodulation of pallidal efferents to the thalamus.

Field of Forel is further away from the internal capsule, and, based on our experience with radiofrequency surgery, sensorimotor responses to FF macrostimulation are rare. The anatomical location of the GPi has several inherent problems, which can suppress optimal clinical effects (Koeglsperger et al., 2019). The posterior limb of the internal capsule is medially located to the GPi, and current spread to the posterior limb of the internal capsule can induce dysarthria, dysphagia, and muscle twitch (Berman et al., 2009; Koeglsperger et al., 2019). The optic tract is located inferior to the GPi, and current spread to the optic tract can induce phosphene (Koeglsperger et al., 2019). Additionally, Parkinsonism, including bradykinesia, postural instability, and gait disturbance also develop by stimulating GPi itself (Zauber et al., 2009; Schrader et al., 2011). The etiology of stimulation-induced Parkinsonism remains unclear. One recent study revealed that the GPi itself could be the region underlying stimulation-induced Parkinsonism (Mahlknecht et al., 2018). Unfortunately, stimulation of the FF induced bradykinesia and postural instability, which suggests that stimulation-induced parkinsonism may originate from neuromodulation of the CBTC itself. In our previous study investigating the radiofrequency ablation of pallidothalamic tract at FF for dystonia and Parkinson’s disease, hypophonia and dysarthria were the most common complications (Horisawa et al., 2021). However, the present study did not show stimulation-induced speech complications. No other stimulation-induced adverse effects were found in this study.

In this study, three patients experienced early improvements of dystonia with relatively lower pulse width stimulation at FF. In this study, we started stimulation on the next day after electrode implantation. Microlesion effects may be attributed to the early improvement of dystonia in Cases 1 and 3, both showing significant improvements at 1 week after surgery. Additionally, the FF target is smaller structures that may require a smaller stimulation field compared to the GPi. Neudorfer et al. (2017) applied FF to the DBS target for Tourette syndrome because the passage of most pallidothalamic fibers through the FF occurs within a diameter < 4 mm (Neudorfer et al., 2017; Neudorfer and Maarouf, 2018). Moreover, axons are more sensitive to electrical stimulation than cell bodies (Vitek, 2002a; Kringelbach et al., 2007). The FF-DBS attempts to stimulate pallidothalamic tract (axons), indicating that relatively narrow field stimulation with lower amplitude and pulse width is possible for the treatment of dystonia. However, its effects remain unclear due to the small sample size in this study.

This was a non-blinded evaluation with a small number of patients and a short follow-up period. The absence of any neuropsychological and cognitive evaluations was also a limitation.

We found that FF-DBS significantly improved dystonia in three patients. Although stimulation-induced complications developed in two patients, this was managed by stimulation adjustments, thus no other stimulation-induced adverse effects were found. Larger sample sizes are needed to further investigate the safety and efficacy of FF-DBS for dystonia.

## Data Availability Statement

The raw data supporting the conclusion of this article will be made available by the authors, without undue reservation.

## Ethics Statement

The studies involving human participants were reviewed and approved by the Ethics Committee of the Tokyo Women’s Medical University. The patients/participants provided their written informed consent to participate in this study.

## Author Contributions

SH: conception and design of the study, acquisition and analysis of data, and drafting a significant portion of the manuscript and figures. KK and MM: acquisition and analysis of data. TK: conception and design of the study. TT: conception and design of the study and acquisition and analysis of data. All authors contributed to the article and approved the submitted version.

## Conflict of Interest

The authors declare that the research was conducted in the absence of any commercial or financial relationships that could be construed as a potential conflict of interest.

## Publisher’s Note

All claims expressed in this article are solely those of the authors and do not necessarily represent those of their affiliated organizations, or those of the publisher, the editors and the reviewers. Any product that may be evaluated in this article, or claim that may be made by its manufacturer, is not guaranteed or endorsed by the publisher.
